# Context-based refinement of mappings in evolving life science ontologies

**DOI:** 10.1186/s13326-023-00294-8

**Published:** 2023-10-19

**Authors:** Victor Eiti Yamamoto, Juliana Medeiros Destro, Julio Cesar dos Reis

**Affiliations:** Institute of Computing, University of Campinas, Campinas, SP, Brazil

**Keywords:** Ontology alignment, Ontology evolution, Mapping refinement, Concept addition, Biomedical vocabulary

## Abstract

**Background:**

Biomedical computational systems benefit from ontologies and their associated mappings. Indeed, aligned ontologies in life sciences play a central role in several semantic-enabled tasks, especially in data exchange. It is crucial to maintain up-to-date alignments according to new knowledge inserted in novel ontology releases. Refining ontology mappings in place, based on adding concepts, demands further research.

**Results:**

This article studies the mapping refinement phenomenon by proposing techniques to refine a set of established mappings based on the evolution of biomedical ontologies. In our first analysis, we investigate ways of suggesting correspondences with the new ontology version without applying a matching operation to the whole set of ontology entities. In the second analysis, the refinement technique enables deriving new mappings and updating the semantic type of the mapping beyond equivalence. Our study explores the neighborhood of concepts in the alignment process to refine mapping sets.

**Conclusion:**

Experimental evaluations with several versions of aligned biomedical ontologies were conducted. Those experiments demonstrated the usefulness of ontology evolution changes to support the process of mapping refinement. Furthermore, using context in ontological concepts was effective in our techniques.

## Introduction

Advancements in biomedical research require relying upon several sources of voluminous, dynamic, heterogeneous, and complex datasets, resulting in difficulties in using and reusing available data. This generates an ever greater demand for adequate computer-supported methods for automatically locating, accessing, sharing, analyzing, and meaningfully integrating data.

Biomedical information systems have intensively relied on semantic technologies such as ontologies to turn the semantics of information explicit for machines. Over the last decade, the biomedical domain has exploited ontologies and their capabilities for various purposes ranging from information retrieval to data management and sharing. However, the size of this domain often requires using several ontologies whose elements are linked through mappings. Mappings are the materialization of semantic relations between elements of interrelated ontologies [1].

Creating mappings between ontologies is a complex task, especially due to the increasing size of biomedical ontologies, which usually contain hundreds of thousands of concepts that need to be interconnected via mappings. Several automatic ontology alignment techniques have been proposed [2]. Nevertheless, significant manual validation efforts are still required to obtain a certain level of quality. This prevents software applications from relying on automatically generated mappings to fully take advantage of them.

Ontologies constantly evolve to reflect the expansion of the knowledge domain under-representation. Ontology changes may affect mappings already established or can be a source for treating mapping refinement. In this context, to avoid the costly ontology re-alignment process, it is crucial to have adequate mapping techniques to keep mappings semantically valid over time [3]. This problem greatly affects the aforementioned large biomedical ontologies, used as information sources for several medical, pharmaceutical, and other related systems. Manual mapping maintenance is possible only if modifications are applied to a restricted number of mappings. Otherwise, reliable automatic methods are required for large and highly dynamic ontologies.

Matching approaches are usually approximations, and the identification of mappings is based on relatedness between concepts. The semantic relations identified during the matching process can be expanded through mapping refinement. Refinement can modify or enrich semantic relations. For instance, during the refinement process, an equivalence ($\equiv$) relation (*i.e.*, a relation defining that two interrelated concepts are equivalent) can be modified to an *is-a* ($\sqsubseteq$) (*i.e.*, representing a relation in which one concept is a specialization of the other) [4].

The challenges of mapping refinement are due to the difficulties in establishing semantic relations between concepts beyond the relatedness identified by traditional matching procedures. In this context, enriched semantic correspondences in ontology mapping might boost ontology merging [5]. In this work, we assume that understanding how concepts were updated over time may be useful to refine the semantic relation in mappings. These changes indicate how concepts and their relationships with each other evolved. This information might support the decision and application of refinement actions intended to modify the type of semantic relation in mappings.

The design of techniques for mapping maintenance according to the different types of ontology changes has been coped within existing approaches. Previous work presented a mapping adaptation strategy for two out of three categories of ontology evolution: *removal* of knowledge and *revision* of knowledge [3]. For example, when concepts are removed, heuristics are designed to apply adaptation actions over mappings automatically. The *addition* of knowledge (third category) is the most frequent type of change occurring in ontology evolution. New concepts are added to comply with new domain knowledge. Such new knowledge needs to be aligned with the already interrelated ontologies. Further investigations are required to benefit from exploiting existing mappings and the contextual information of neighbor concepts for mapping refinement based on new concepts added. In the present study, we focused only on the creation of mapping based on the addition of knowledge.

In this paper, we propose mapping refinement methodologies to update mapping sets taking ontology changes into account (based on new concepts added in ontology evolution). In our proposals, we study the use of conceptual information related to neighbor concepts and the ontology changes for enhancing mapping completeness. For this purpose, we investigate two distinct techniques: 1) reuse of already established mappings by exploring the role of neighbor concepts to suggest new mappings to align new concepts in ontology evolution; and 2) ontology changes as a valuable source of information to enhance the correspondences found between concepts beyond equivalence. Our proposal allows suggesting new correspondences without applying a matching operation with the whole set of ontology entities.

Our experimental evaluation explored real-world biomedical ontologies and mappings established between them. We examine the quality of the automatically-suggested enriched set of mappings concerning the set of new correspondences observed in the official updated release of mappings via standard evaluation metrics. Our study reveals a promising approach to using ontology evolution changes, particularly addition operations, to enhance semantic relations in mappings. Besides, the results show innovative findings regarding how mappings can be refined based on new concepts added. We demonstrate that local matching considering neighbor concepts is competitive with a matching operation with the whole target ontology.

This article expands on previous studies conducted by the authors. The study performed by Destro et al. [6] focused on the cross-language refinement problem. It was revisited and applied to monolingual ontology mappings. It was combined with the proposed mapping refinement methodology described by Yamamoto et al. [7]. This work contributes with techniques that enable turning ontology mapping sets further adequate based on ontology changes and analysis of previous and novel ontology release versions.

The remainder of this article is organized as follows: Section “Background” presents the background by describing the synthesis of a literature review; Section “Methods” describes the problem formalization and research questions of the conducted studies; Section “Analysing new ontology version to refine mappings” reports on our study to refine ontology mappings for newly added concepts in a novel ontology version; Section “Analysing old ontology version to refine mappings” describes our proposal and evaluation for refining ontology mappings relying on an existing old version of the ontology and changes derived in the ontology evolution. Section “Discussion” discusses the obtained findings. Section “Conclusion” draws conclusions and future work.

## Background

Previous studies have investigated semi-automatic approaches to adapt ontology mappings when at least one of the mapped ontologies evolves [8]. In this direction, Dos Reis et al. conceptualized the *DyKOSMap* framework [3] to support the adaptation of ontology mappings. This framework highlighted different aspects by including the role of different types of ontology changes and considering the conceptual information related to established mappings. Adaptation of ontology mappings usually relies on ontology changes computation. In this context, Hartung et al. proposed the *Conto-Diff* software tool [9] for the automatic detection of simple and complex ontology changes.

Some techniques have used external resources aiming to improve and increase the number and precision of established mappings. Stoutenburg [10] argued that the use of upper ontologies (an ontology that consists of common general terms across domains) and linguistic resources could enhance the alignment process.

Other approaches have combined lexical-based and semantic-based algorithms, mostly using resources available in the Unified Medical Language System (UMLS)[11] for generating mappings. The use of UMLS as an external resource can be interesting in various aspects: (1) favors an increase in the number of mapping; (2) provides different synonyms terms for a given concept; and (3) defines relations between concepts in a semantic network. Zhang and Bodenreider [12] explored UMLS to improve alignment between anatomical ontologies. They showed that domain knowledge is a key factor for the identification of additional mappings compared with the generic schema-matching approach. However, Nadkarni et al. raised that UMLS needs a selection of subset to be used for matching. Additionally, concepts from sources like ICD-9 and ICD-10 have a problem with highly composite concepts that are similar but differentiated by the presence or negation of one or more terms [13].

Sekhavat and Parsons [14] explored conceptual models (e.g., Entity Relationship, Class diagrams or domain ontologies) as background knowledge to enrich database schema mappings and resolve ambiguous mappings. Their approach used conceptual models as external resources to capture the semantics of schema elements, for instance, a pair of concepts $c_1$ and $c_2$ where $c_1$ is a subclass, and $c_2$ is a superclass in a conceptual model. This information enriched the schema before mapping, marking the foreign keys corresponding to $c_1$ and $c_2$ as generalizations. As a consequence, the relationship identified in the schema mapping is a generalization (*is-a*) instead of equivalence.

Pruski et al. [15] proposed exploiting domain-specific external sources of knowledge to characterize the evolution of concepts in dynamic ontologies. The technique analyzed the evolution of values in concept attributes. The approach used ontological properties and mappings between ontologies from online repositories to deduce the relationship between a concept and its successive version.

Noy et al. [16] and Seddiqui et al. [17] explored anchor concepts to obtain mappings. They used a set of concept pairs aligned to obtain other mappings based on these pairs. These approaches calculated new alignment for all concepts from the involved ontologies, but they are not used for ontology evolution.

Ontology mapping refinement helps expand the types of semantic relations identified during the matching process and relies on previously calculated ontology mappings. The main approaches available in the literature for refinement are based on external resources or manual pattern definition. In this context, *TaxoMap* [18] refers to an approach that brought together mapping and refinement by using *WordNet* [19] lexical database as background knowledge and explored pattern-based refinement techniques. TaxoMap uses manually created patterns to refine mappings in the same domain. In contrast, Spiliopoulos et al. [20] presented the *Classification-Based Learning of Subsumption Relations* method for ontology alignment. This automated method explores patterns describing the relation between concepts (e.g., attributes with the same content). Machine learning methods are applied to help identify these patterns.

The work conducted by Arnold and Rahm [4] defined a mapping refinement technique by using a set of equivalent mappings as input. They explored generic external resources and proposed a two-step enrichment technique to improve existing imprecise mappings by using linguistic techniques and resources like *WordNet* to refine semantic relations between aligned concepts. Their objective was to transform equivalence between concepts into an *is-a* or *part-of* relation, which may further reflect the real semantics of mapped concepts. Although as an approach commonly explored, external resources influence the results and need further research to determine their impact.

Yamamoto et al. [7] proposed a method to create new mappings for newly added concepts identified during an ontology evolution without matching the entire ontology again. However, this method is limited to equality relationships and only analyze newly added concepts. Destro et al. [6] proposed a refinement technique to update the semantic mapping type by using information obtained from ontology evolution. The algorithm identifies if the ontology change operation is an addition of a new concept or revision of existing concepts and applies the necessary operation to modify or enrich the semantic relation. In this paper, we present the two approaches for monolingual case.

The use of ontology evolution in mapping refinements require further investigation in the literature. This work explores two methods using ontology change operations to leverage refinement, particularly concept addition. The approaches proposed by Yamamoto et al. and Destro et al. differ from the other mentioned proposals because they rely only on ontology change operations, information obtained from the ontology itself, without depending on external tools or resources. The algorithm leverage the information obtained from ontology change operations to identify refinement actions applicable to mappings. To the best of our knowledge, this approach has not been investigated in the literature before.

We demonstrate how the evolution of concepts can be useful to enrich the semantics of correspondences already established. We also contribute with a methodology to consider newly added concepts and investigate the context of candidate target concepts of existing mappings for refinement over time. We further evaluate the proposed algorithms by measuring the effectiveness of mapping refinement approach on real-world biomedical ontologies.

In the next section, the methodologies supporting our studies and claims are described.

## Methods

Subsection “Formal definitions” presents the formal definitions useful to this investigation. Subsection “Methodology” describes the conducted methodology by defining the studies and open research questions investigated in this work.

### Formal definitions

**Ontology.** An ontology $\mathcal {O}$ specifies a conceptualization of a domain in terms of concepts, attributes and relationships [21]. Formally, an ontology $\mathcal {O} = (\mathcal {C}_\mathcal {O}, \mathcal {R}_\mathcal {O}, \mathcal {A}_\mathcal {O})$ consists of a set of concepts $\mathcal {C}_\mathcal {O}$ interrelated by directed relationships $\mathcal {R}_\mathcal {O}$. Each concept $c \in \mathcal {C}_{\mathcal {O}}$ has a unique identifier and is associated with a set of attributes $\mathcal {A}_\mathcal {O}(c)=\{a_1, a_2, ..., a_p\}$. Consider attributes as string terms characterizing the meaning of concepts. Each relationship $r(c_1, c_2) \in \mathcal {R}_\mathcal {O}$ is typically a triple $(c_1, c_2, t)$ where *t* is the relationship (e.g., *is_a*, *part_of*, *adviced_by*, *etc.*) interrelating $c_1$ and $c_2$.

**Context of a concept.** We define the context of a particular concept $c_i \in \mathcal {C}_\mathcal {O}$ as a set of *super concepts*, *sub concepts* and *sibling concepts* of $c_i$, as following:

1 $$\begin{aligned} CT(c_i, \lambda ) = sup(c_i, \lambda ) \cup sub(c_i, \lambda ) \cup sib(c_i, \lambda ) \end{aligned}$$

where

2 $$\begin{aligned} sup(c_i, \lambda )= & {} \{c_j|c_j \in \mathcal {C}_\mathcal {O}, r(c_i, c_j)=``\sqsubset " \wedge length(c_i, c_j) \le \lambda \wedge c_i \ne c_j \} \nonumber \\ sub(c_i, \lambda )= & {} \{c_j|c_j \in \mathcal {C}_\mathcal {O}, r(c_j, c_i)=``\sqsubset " \wedge length(c_i, c_j) \le \lambda \wedge c_i \ne c_j \} \nonumber \\ sib(c_i, \lambda )= & {} \{c_j|c_j \in \mathcal {C}_\mathcal {O}, ( (sup (c_j) \cap sup (c_i)) \vee (sub (c_j) \cap sub (c_i)) ) \nonumber \\{} & {} \wedge length(c_i, c_j) \le \lambda \wedge c_i \ne c_j \} \end{aligned}$$

where $\lambda$ is the level of the context. It represents the maximum value for the length between two concepts (in terms of their shortest relationship distance in the hierarchy of concepts) and the “$\sqsubset$” symbol indicates that “$c_i$ is a sub concept of $c_j$”. This definition of $CT(c_i, \lambda )$ is specially designed as the relevant concepts to be taken into account in the settings of this investigation on mapping refinement.

**Similarity between concepts.** Given two particular concepts $c_i$ and $c_j$, the similarity between them is defined as the maximum similarity between each couple of attributes from $c_i$ and $c_j$. Formally:

3 $$\begin{aligned} sim(c_i, c_j) = \arg \max ~sim(a_{ix}, a_{jy}) \end{aligned}$$

where $sim(a_{ix}, a_{jy})$ is the similarity between two attributes $a_{ix}$ and $a_{jy}$ denoting concepts $c_i$ and $c_j$, respectively.

**Mapping.** Given two concepts $c_s$ and $c_t$ from two different ontologies, a mapping $m_{st}$ can be defined as:

4 $$\begin{aligned} m_{st} = (c_s, c_t, semType, conf) \end{aligned}$$

where *semType* is the semantic relation connecting $c_s$ and $c_t$. In this article, we differentiate *relation* from *relationship*, where the former belongs to a mapping and the latter to an ontology. The following types of semantic relation are considered: *unmappable* [$\bot$], *equivalent* [$\equiv$], *narrow-to-broad* [$\le$], *broad-to-narrow* [$\ge$] and *overlapped* [$\approx$]. For example, concepts can be equivalent (*e.g.*, *head*$\equiv$*head*), one concept can be less or more general than the other (*e.g.*, *diabetes type I*$\le$*diabetes*) or concepts can be somehow semantically related ($\approx$). The *conf* is the similarity between $c_s$ and $c_t$ indicating the confidence of their relation [22]. We define $\mathcal {M}_{ST}$ as a set of mappings $m_{st}$ between ontologies $\mathcal {O}_S$ and $\mathcal {O}_T$.

**Versions of ontology and mappings.** At a given time $j \in N$, we assume the version of an ontology release $\mathcal {O}_S^j$. For instance, ontology $\mathcal {O}_S^0$ is version 0 whereas $\mathcal {O}_S^1$ is version 1 of the same ontology. In another sense, $\mathcal {O}_S^{j+1}$ is the new version of the $\mathcal {O}_S^{j}$. Similarly, we consider $\mathcal {M}^{j}_{ST}$ a release of a set of mappings between two ontologies, such that, $\mathcal {M}^{j+1}_{ST}$ is the new version of the mappings produced.

**Ontology change operations (OCO).** An ontology change operation (OCO) is defined to represent a change in an attribute, in a set of one or more concepts, or in a relationship between concepts. OCOs are classified into two main categories: *atomic* and *complex* changes. Each OCO in the former cannot be divided into smaller operations while each one of the latter is composed of more than one atomic operation. In this work, we pay further attention to the operations of concept addition.

### Methodology

This work conducts two studies investigating ontology refinement phenomena under different perspectives. Ontologies used in both studies have multiple versions available. This is a *sine qua non* condition to apply the techniques described in this work.

**Analysing new ontology version to refine mappings.** (*cf*. Section “Analysing new ontology version to refine mappings”). Consider two versions of the same source ontology $\mathcal {O}_S^j$ at time *j* and $\mathcal {O}_S^{j+1}$ at time ${j+1}$, a target ontology $\mathcal {O}_T^j$, and an initial set of mappings $\mathcal {M}^j_{ST}$ between $\mathcal {O}_S^j$ and $\mathcal {O}_T^j$ at time *j*. Suppose that the frequency of new releases of $\mathcal {O}_S$ and $\mathcal {O}_T$ is different and at time $j+1$ only $\mathcal {O}_S$ evolves. It is necessary to refine $\mathcal {M}^j_{ST}$ to guarantee the quality and completeness of $\mathcal {M}^{j+1}_{ST}$ according to the new concepts of the ontology version. We aim to obtain $M^{j+1}_{ST}$ based on the original mapping set $M^{j}_{ST}$ between the ontologies $\mathcal {O}_S^j$ and $\mathcal {O}_T^j$. At time $j+1$, newly added concepts appear in $\mathcal {O}_S^{j+1}$ and we attempt to refine the original mapping set $M^{j}_{ST}$ to provide a set of valid mappings $M^{j+1}_{ST}$.

We study how $\mathcal {M}^j_{ST}$ can be refined (e.g., new mappings derived) based on ontology changes related to *addition of knowledge*. To this end, our work addresses the following research questions:

- How can existing mappings be exploited for mapping refinement based on added new concepts?
- Is it possible to reach mapping refinement for the alignment of new concepts without applying a matching operation in the whole target ontology?
- What is the impact of using the context of concepts $CT(c_i, \lambda )$, including evolution information, in both source and target ontologies on the mapping refinement effectiveness?

**Analysing old ontology version to refine mappings.** (*cf*. Section “Analysing old ontology version to refine mappings”) Consider two versions of the same source ontology $\mathcal {O}_S^{j-1}$ at time $j-1$ and $\mathcal {O}_S^j$ at time *j*. Note that $\mathcal {O}_S^j$ is the current ontology under use, and $\mathcal {O}_S^{j-1}$ refers to an old version of the same ontology. In this study, it is useful to understand how a given concept in $\mathcal {O}_S^j$ has evolved from $\mathcal {O}_S^{j-1}$. In our problem modeling, there is a target ontology $\mathcal {O}_T^j$, and a set of mappings $\mathcal {M}^j_{ST}$ between $\mathcal {O}_S^j$, and $\mathcal {O}_T^j$ at time *j*. We suppose that the frequency of new releases of $\mathcal {O}_S$ and $\mathcal {O}_T$ is different, and at time *j*, only ${O}_S$ has evolved. We assume that concepts added by the evolution will likely provide useful information for mapping refinement of $\mathcal {M}^j_{ST}$. We aim to analyze previous concept information (in the old ontology version – at time $j-1$) to enrich semantic relations in mappings and obtain the refined mapping set $\mathcal {M'}_{ST}^j$ at time *j*. All mappings in $\mathcal {M}^j_{ST}$ have initially the type of semantic relation *equivalent* [$\equiv$] or *overlapped* [$\approx$], and we assume them as a mapping candidate set.

In this problem, given a mapping $m_{12} \in \mathcal {M}^j_{ST}$ associated with a concept $c_1$ affected by changes in the ontology, the challenging issue is to determine an exact and suited action of refinement to apply to $m_{12}$. To address this challenge, we define and formalize a set of *mapping refinement actions* (*cf*. Section “Analysing old ontology version to refine mappings”). The mapping refinement actions are part of refinement procedures, playing a key role in improving the quality of mappings. The objective is to enrich the mapping set by considering different semantic relations between concepts. For instance, equivalence relations are refined to *is-a* or *part-of*.

We aim to obtain $\mathcal {M}'^{j}_{ST}$, a refined mapping set based on the input original mapping set $\mathcal {M}^{j}_{ST}$ (already produced and given as input to our technique). We refine mappings in $\mathcal {M}^{j}_{ST}$ based on new concepts added to $\mathcal {O}_S^j$ when compared to $\mathcal {O}_S^{j-1}$. In particular, we address the following research questions:

- How to apply mapping refinement actions for deriving mappings based on changes concerning the addition of concepts?
- How to modify the type of semantic relation in mapping observing past release versions of the ontology?
- How to explore the context of concepts $CT(c_i, \lambda )$ (neighborhood) in mapping refinement attempting to benefit from a local re-matching in the procedure?

#### Analysing new ontology version to refine mappings

We aim to propose adequate correspondences for each newly added concept at time $j+1$. In the first step, our approach identifies all newly added concepts using the *Conto-Diff* tool [9]. This tool allows the identification of atomic and complex ontology changes. Next, we extract the contextual information, *i.e.*, super, sub, and sibling concepts of those newly added concepts (*cf.* Formula 1). It is important to distinguish between contextual information and linguistic context. While contextual information represents the neighborhood of the concept, linguistic context refers to the surrounding words and phrases that provide clues to the meaning of a concept. In this method, we used the first definition. We then examine the existing mappings between the source concept in the context of the newly added concept and the corresponding target concepts. The idea behind the context-oriented technique is that the candidate mapping is established between a newly added concept and a target concept of an existing mapping at time *t*.

The proposed method is based on three main parameters: source level, target level, and threshold. The source level defines the maximum distance between the newly added concept and the concept to be explored in the source ontology. The target level defines the maximum distance between the concept with mapping in the previous version and the candidate concept in the target ontology (*cf.* Formula 1). Threshold defines the minimum similarity value between two concepts to create a new mapping.

**Fig. 1:**
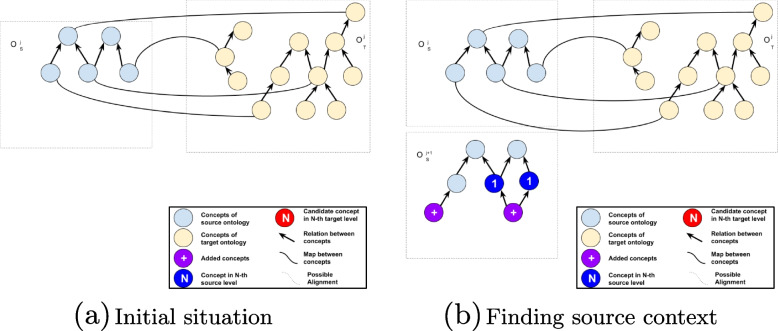
Situations before applying alignment algorithm. Adapted from [7]

Figure 1 (a) illustrates a situation where two ontologies are presenting an alignment in time *j*. Figure 1 (b) illustrates a situation where source ontology evolves and some concepts are added to the ontology source at time $j+1$ (new concepts added). The algorithm finds newly added concepts and explores the context of each newly added concept. Source level is the maximum distance between the newly added concept and the concept to be explored. In this example, the context of the right concept is explored using source level 1.

**Fig. 2:**
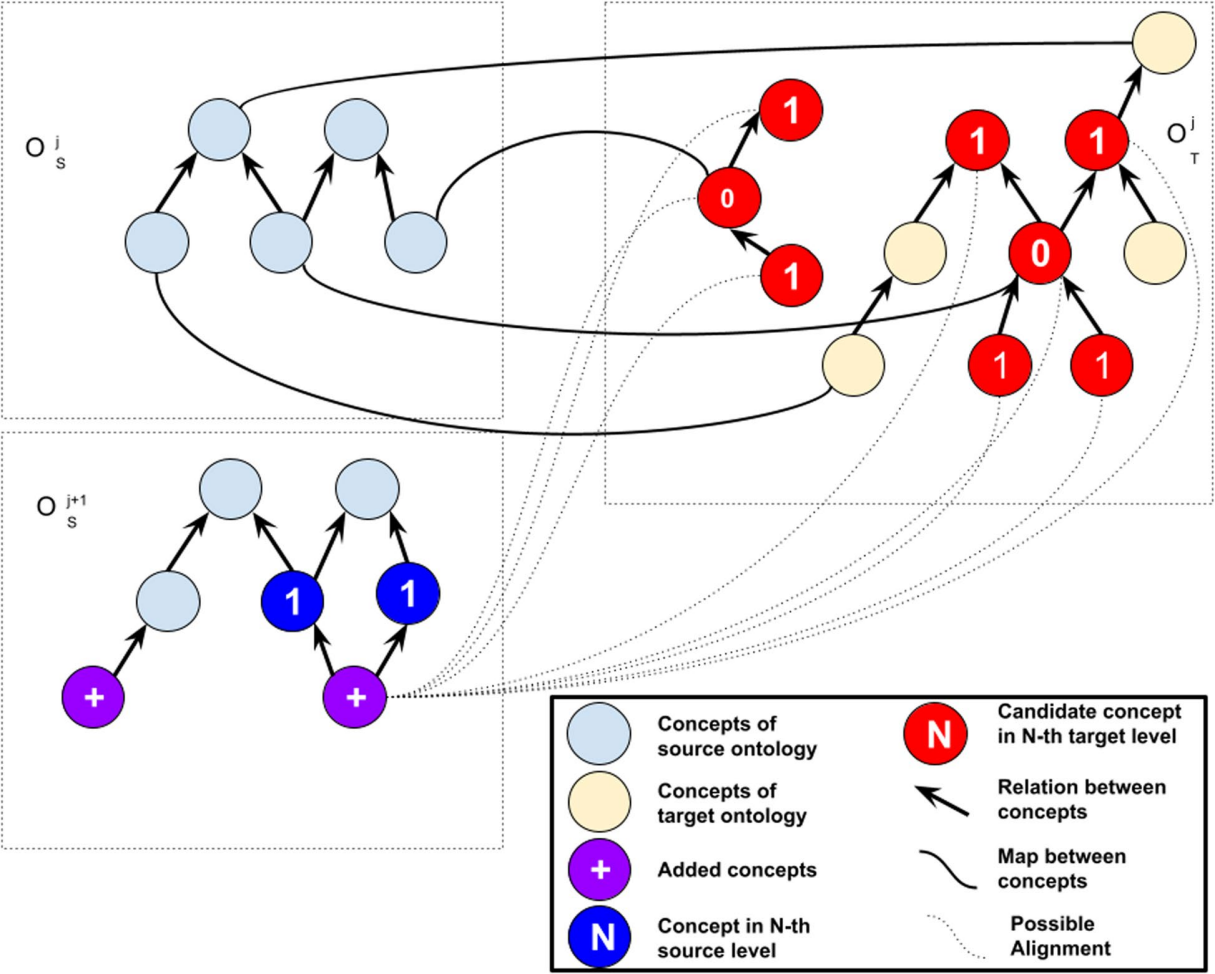
Calculating similarity with candidate concepts. Adapted from [7]

After finding concepts inside the context of newly added concepts that align at time *j*, the aligned concepts from target ontologies are added as candidate concepts. The context of each aligned concept in the target ontology is explored and added as candidate concepts. Target level is the maximum distance between the aligned concept and the candidate concept in the target ontology. Figure 2 illustrates this situation using target level 1. The number of candidates depends on how dense the ontology is. The denser and more connected, the higher the number of candidate concepts.

Algorithm 1 requires a source ontology in time j, source ontology in time j + 1, target ontology in time j, mapping between source and target ontology in time j, a natural number $\gamma$ as source level, a natural number $\lambda$ as a target level and a real number $\tau$ defining the threshold. The source level, target level, and threshold can be defined by the user. The algorithm computes the difference between two given versions of the source ontology (line 1). For each newly added concept $c_i^{j+1}$, the algorithm considers a candidate concept, namely $c_t^{j}$ in the target ontology by exploiting already existing mappings related to $CT(c_i^{j+1}, \gamma )$ (lines 4-8). This algorithm explores information from the past version of the ontology to refine new mapping for the newer version. Hence, the mapping used to explore is recovered from the previous evolution version ($c_k^{j}$) of the concept $c_k^{j+1}$ found in the context of $c_i^{j+1}$.

For each $c_t^{j}$, the algorithm obtains a set of concepts from $CT(c_t^{j}, \lambda )$. We determine a new refined mapping by calculating the similarity between a new concept $c_i^{j+1}$ of $\mathcal {O}^{j+1}_S$ and a candidate $c_n \in \mathcal {C}_t$. Algorithm 1 searches for the candidate $c_t$ that yields the maximum similarity value. To calculate the similarity between those concepts, each attribute of the evaluated concept is compared with attributes in candidate concepts. The best similarity value obtained between those attributes is defined as the similarity between two concepts. If the maximum similarity among attributes of the concept is greater than or equal to a threshold $\tau$, the algorithm establishes a mapping between the newly added concept and the candidate target concept (lines 10-18).

**Figure:**
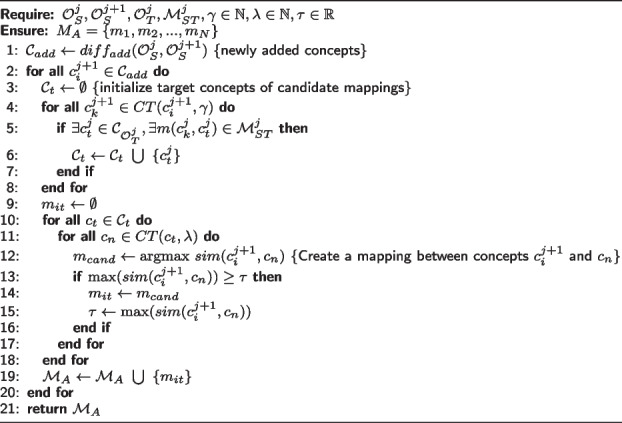
**Algorithm 1** Contextual approach to mapping refinement

In order to compare with the results obtained by our approach, we propose another algorithm that ignores new concepts’ context to calculate similarity. It means that the algorithm computes the similarity between each newly added concept with all concepts in the target ontology.

Algorithm 2 computes the difference between two given versions of the source ontology (line 1). For each newly added concept, the algorithm compares the concept with all concepts from the target ontology using similarity computed with concept attributes. If the maximum similarity is greater than a given threshold $\tau$, a new mapping is created between the newly added concept and the concept from the target ontology with the best similarity (lines 2-13).

**Figure:**
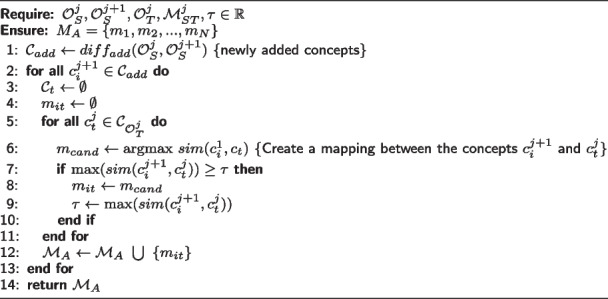
**Algorithm 2** All concepts approach mapping refinement

In our algorithms, the attributes of each source concept are compared with attributes of all target concepts to obtain similarity value between concepts. The value of similarity between two concepts is the maximum value of similarity from their attributes.

Let *n* be the quantity of newly added concepts and *m* the quantity of all concepts in the target ontology. Algorithm 1 computes the similarity of each newly added concept and all candidate concepts in the target ontology. The size of candidate concepts for each added concept varies by the source level and target level used. If the source level and target level are high enough, the number of candidates can be approximated by m, resulting in time complexity of $O(n \times m)$. However, the source level and target level explored in this research used low numbers. For this reason, the size of candidate concepts is approximated as a constant resulting in time complexity *O*(*n*). Algorithm 2 computes the similarity of each newly added concept and all concepts in the target ontology, consequently, the time complexity is $O(n \times m)$.

#### Analysing old ontology version to refine mappings

In this section, we define mapping refinement actions and design algorithms to use them as pre-defined behavior to enrich ontology mappings according to ontology evolution.

**Fig. 3:**
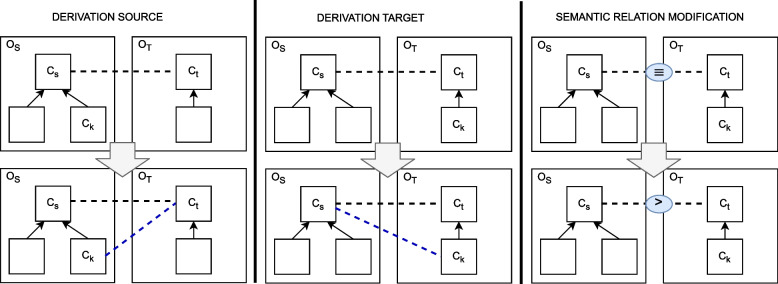
Mapping refinement actions. New mappings added by actions are represented by dashed blue lines. Note: Reprinted from [6]

Three distinct actions for refining mappings are defined (*cf*. Fig. 3): *derivation of source concept*, *derivation of target concept* and *semantic relation modification*. In the following, we formally describe each action. To this end, let $m_{st} \in \mathcal {M}_{ST}^j$ be the mapping between two particular concepts $c_s \in \mathcal {O}_S^j$ and $c_t \in \mathcal {O}_T^j$.

**Mapping derivation source:** an existing mapping from $\mathcal {M}_{ST}^j$ derives a new mapping with the same target concept and different source concept. This action results in the addition of a new mapping $m_{kt}$ to $\mathcal {M}'^j_{ST}$.

5 $$\begin{aligned} \begin{array}{l} deriveS(m_{st},c_k)\longrightarrow m_{st}\in \mathcal {M}^j_{ST} \wedge m_{kt}\notin \mathcal {M}^j_{ST} \wedge \\ (\exists c_k \in \mathcal {O}_S^j, m_{kt} \in \mathcal {M}'^j_{ST}\wedge sim(c_s,c_k)\ge \sigma ) \wedge \\ m_{st} \in \mathcal {M}'^j_{ST} \end{array} \end{aligned}$$

where $sim(c_s,c_k)$ denotes the similarity between $c_s$ and $c_k \in CT(c_s, \gamma )$ (neighborhood), and $\sigma$ denotes the threshold used to compare the derived mapping.

**Mapping derivation target:** an existing mapping $m_{st}$ in $\mathcal {M}^j_{ST}$ derives a new mapping with the same source and a different target, resulting in the addition of a new mapping $m_{sk}$ to $\mathcal {M}'^j_{ST}$.

6 $$\begin{aligned} \begin{array}{l} deriveT(m_{st}, c_k)\longrightarrow m_{st}\in \mathcal {M}^j_{ST} \wedge m_{sk}\notin \mathcal {M}^j_{ST} \wedge \\ (\exists c_k\in \mathcal {O}_T^j, m_{sk}\in \mathcal {M}'^j_{ST}\wedge sim(c_s,c_k)\ge \sigma ) \wedge \\ m_{st} \in \mathcal {M}'^j_{ST} \end{array} \end{aligned}$$

Where $c_k \in CT(c_t, \gamma )$ represents the neighborhood of the concept in the target ontology.

**Semantic relation modification:** the type of the semantic relation of a given mapping is modified. This action is designed for supporting the refinement of mappings with different types of semantic relations rather than only considering the type of equivalence relation ($\equiv$). The action can be applied simultaneously with the actions of the derivation of mappings. When deriving a mapping, it is also possible to modify the type of semantic relation of such mapping.

7 $$\begin{aligned} \begin{array}{l} modSemType(m_{st}, new\_semType_{st})\longrightarrow m_{st} \in \mathcal {M}'^j_{ST} \wedge \\ new\_semType_{st} \in \{ \bot , \equiv , \le , \ge , \approx \} \\ \wedge semType_{st} \ne new\_semType_{st} \end{array} \end{aligned}$$

In the mapping refinement phase, concepts from two versions of the source ontology ($\mathcal {O}_S^{j-1}$ and $\mathcal {O}_S^j$) are taken into account to refine a candidate mapping set. The necessary instances of ontology change operations are identified from one ontology version at time $j-1$ to another at time *j* with a comparison computation procedure [9]. It generates a set of changes identified between two versions of the same ontology. The change history of the ontology, provided by the authors or curators of the ontology, may also be used if available. In this article, we only consider the newly added concepts from version $\mathcal {O}_S^{j-1}$ to $\mathcal {O}_S^j$.

As input for our mapping refinement procedure, we consider a set of input mapping set candidates $\mathcal {M}^j_{ST}$. In this sense, our procedure is not responsible for creating the initial whole set of mappings. We describe the mapping refinement procedure in two phases:

1. Either the change history or the output of executed ontology change detection tools is used to identify mappings with the potential of refinement, based on the type of ontology evolution operations affecting the concepts in $\mathcal {O}_S^{j}$. For instance, the addition of a concept to an ontology may indicate a specialization of another concept (e.g., the concept *Eagle* in $\mathcal {O}_S^{j}$ was added as a child of the concept *Bird*, being the former a specialization of the latter). Therefore, any candidate mapping involving the concepts *Eagle* and *Bird* are identified with the possibility of refinement.
2. After the selection of mappings for refinement, for each selected mapping from $\mathcal {M}_{ST}^{j}$, an action is executed based on the type of ontology change. The action may include a direct decision to perform modification in the semantic relation of the candidate mapping (e.g., a $\equiv$ relationship may be replaced with a $\sqsubseteq$), or other appropriate action. This work emphasizes the concept of addition operation in ontology evolution. In this sense, all candidate mappings in $\mathcal {M}_{ST}^{j}$ related to a newly added concept from $\mathcal {O}_S^{j-1}$ are subject of refinement by our technique.

**AdditionProcedure.** This procedure is invoked when $c_s$ is a new concept added to $\mathcal {O}_S^j$. Algorithm 3 presents the proposed approach to refining mappings associated with the addition of concepts changes. For each mapping, $m_{st}$, the neighborhood of both $c_s$ and $c_t$ is retrieved to perform a local rematch. The *rematch* function receives a set of source concepts $C_s$ and a set of target concepts $C_t$ and returns a similarity matrix (*simMatrix*). The objective of applying a local rematch is to compare the similarities between the neighborhood of the source and target concepts.

The similarity values found drive modifications to the semantic relation established in $m_{st}$. For example, if $sim(sup(c_s), c_t) > sim(c_s, c_t)$, the algorithm modifies the semantic relation in $m_{st}$ to the same semantic relation of $sup(c_s) \text { and } c_t$; and add a new mapping between $sup(c_s)$ and $c_t$. The local rematch helps establish a derivation of mapping when the $sim(c_s, sub(c_t)) \ge sim(c_s, c_t)$ or $sim(c_s, sup(c_t)) \ge sim(c_s, c_t)$.

**Figure:**
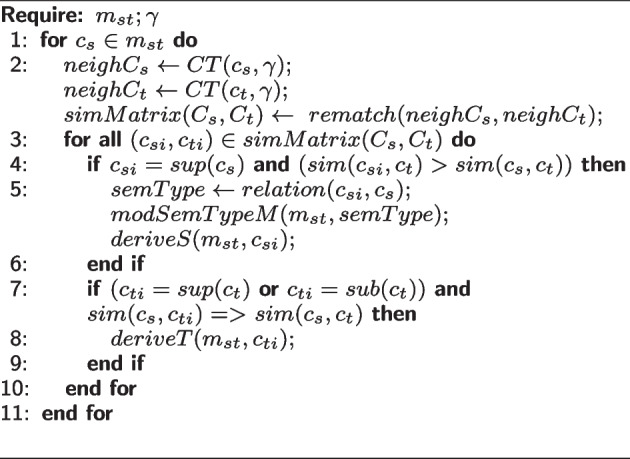
**Algorithm 3** Mapping refinement for additional changes

**Fig. 4:**
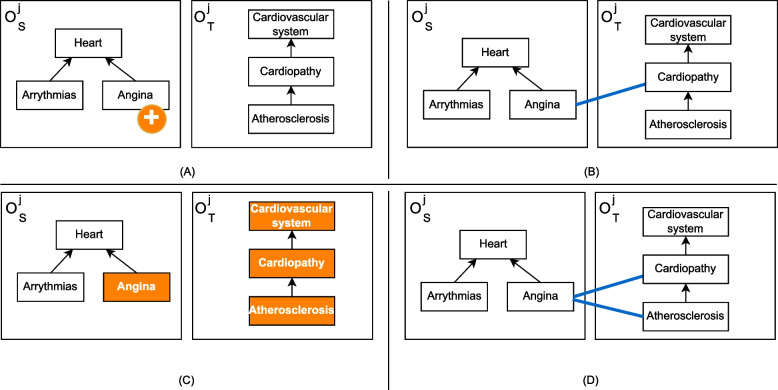
**A** Ontology change operations (OCO) on $\mathcal {O}_S$. **B** Illustration of the mapping $m_{st} \in \mathcal {M}^{j}_{ST}$ candidate for refinement. **C** Computing similarity values between $c_s$ and the $CT(c_t, \gamma )$ (neighborhood). **D** Resulting $\mathcal {M}'^{j}_{ST}$ after our refinement procedure (application of the derivation target action). Note: Reprinted from [6]

We present an example to illustrate the AdditionProcedure. Ontology $\mathcal {O}_S$ evolved over time by generating different versions from time $j-1$ to time *j*, as illustrated by Fig. 4(A). A set of candidate mappings $\mathcal {M}^{j}_{ST}$ between $\mathcal {O}_S^{j}$ and $\mathcal {O}_T^{j}$, at time *j*, is given as input for the refinement procedure. Figure 4(B) illustrates the mapping $m_st \in \mathcal {M}^{j}_{ST}$ between concepts $c_s$ *Angina* and $c_t$ *Cardiopathy*. The refinement procedure requires as input the list of newly added concepts detected from one version of the source ontology to another. Similarity values between concept $c_s$ *Angina* and the concepts of the neighborhood of the target concept *Cardiopathy* at time *j* are calculated via local rematch (*cf*. Fig. 4(C)). If the similarity value between the concepts $c_s$ *Angina* and some neighbor $c_{ti}$ of $c_t$ is higher than the original similarity value given by $sim(c_s,c_t)$, *i.e.* $sim(c_s,c_{ti}) \ge sim(c_s,c_t)$, the algorithm derives a mapping between $c_s$ and $c_{ti}$ to reflect this finding (*cf*. Fig. 4(D)).

For evaluation purposes, we considered an existing mapping set between Logical Observation Identifiers Names and Codes (*LOINC*) ontology published in English and its linguistic variant in Spanish. *LOINC* provides a standard for identifying clinical information (laboratory and clinical test results) in electronic reports [23].

This dataset was chosen because LOINC is freely available, widely used in 176 countries^1^ and presents a regular update schedule of twice a year, providing a number of ontology changes in every new version available. Any matching system may be used for the rematch phase. We used a cross-language matching system available from previous studies [24].

LOINC authors provide the updated changes in the ontology entities in every release in a separate document, specifying the change operations undergone by the entities. The version selected for this evaluation was the 2.65, released in December 2018. The English variant of LOINC contains 89,271 entities, and the Spanish variant contains 54,599 entities.

Our proposed technique requires an initial mapping set as input. For this purpose, we used the existing mapping set between the two linguistic variants of *LOINC* (one ontology in English and the other in Spanish). Each entity has a unique permanent identifier named *LOINC code* (in the sense that it cannot be reused even if the entity is deprecated). This code is invariable across linguistic variants. We use LOINC code to identify equivalent entities between the two selected ontologies. In particular, we focused our evaluation on new concept additions actions.

## Results

### Analysing new ontology version to refine mappings

We aim to validate the quality of the refined set of mappings as the outcome of our approach. Data used in this evaluation come from five biomedical ontologies: SNOMED-CT (SCT), MeSH, ICD-9-CM, ICD10-CM and NCI Thesaurus. SNOMED-CT (Systematized Nomenclature of Medicine-Clinical Terms) is an ontology with the objective of creating a taxonomy of terms referring to the medical domain and a framework of rules guaranteeing that each term is used with exactly one meaning[25]. MeSH Thesaurus is a controlled vocabulary produced by the National Library of Medicine and used to index, catalog and search information and documents related to biomedicine and health^2^ ICD-9-CM and ICD-10-CM are a classification of diseases published by the World Health Organization [26]. NCI Thesaurus^3^ contains terminologies used in the semantic infrastructure and information systems by National Cancer Institute [27]. Table 1 shows the statistics of source and target ontologies for each of the considered versions.

**Table 1:** Statistics of ontologies

Ontology	Release	#Concepts	#Attributes	#Subsumptions	#New Concepts	% of new concepts
4emICD9	2009	12,734	34,065	11,619	325	2.80%
	2011	13,059	34,963	11,962		
ICD10	2011	43,351	87,354	40,330	0	0
SCT	2010	386,965	1,531.288	523,958	8,381	1.60%
	2012	395,346	1,570,504	539,245		
NCI	2009	77,448	282,434	86,822	17,284	19.9%
	2012	94,732	365,515	105,406		
MeSH	2012	50,367	259,565	59,191	604	1.02%
	2013	50,971	264,783	59,844		

Note: Reprinted from [7]

The mappings obtained by the proposed algorithm 1 (Ref. 3.2.1) are compared with the official mappings (their new official release). Mappings between SNOMEDCT and ICD-9-CM are offered by the International Health Terminology Standards Development Organisation (IHTSDO)^4^. Mappings between MeSH and ICD-10-CM were offered by the Catalogue et Indexation des Sites Médicaux de langue Française (CISMeF)^5^. Table 2 shows the quantity of each mapping set between the ontologies used in this experiment.

**Table 2:** Statistics of the studied mappings

SCT-ICD9	#Mappings	SCT-NCI	#Mappings	MeSH-ICD10CM	#Mappings
2010-2009	84,519	2009-2009	19,971	2012-2011	4,631
2012-2011	86,638	2012-2012	22,732	2013-2011	5,378

Note: Reprinted from [7]

To analyze results obtained experimentally, it was necessary to compare our obtained mappings with mappings created only for newly added concepts in the new version of the considered ontologies. Table 3 shows the number of mappings really considered in the metrics.

**Table 3:** Number of new mappings created and associated with newly added concepts in the new ontology version (considered the gold standard)

Mappings	#official mappings created after newly added concepts	Percentage of mappings
SNOMEDCT-ICD9CM	1,583	1.87%
SNOMEDCT-NCI	158	0.7%
MeSH-ICD10CM	21	0.45%

Note: Adapted from [7]

The experiments were performed for three datasets (SCT-NCI, SCT-ICD9 and MeSH-ICD10) considering SCT and MeSH as source ontologies. As assessed configurations, we considered three source levels, three threshold values (0.5, 0.75, and 0.9), and four target levels. For each dataset, we fixed source level and threshold to verify the results for each target level. After examining all target levels, we changed the threshold and repeated for each target level. After examining all threshold values, we changed the source level and repeated the whole procedure for all thresholds and target levels.

The method used to calculate similarity may affect the results. In our proposed algorithms, we explored Bi-gram Dice to calculate similarity. Bi-gram is a sequence of two adjacent letters of a word. The dice coefficient is defined as twice the number of common elements divided by the sum of each element. The Formula 8 shows the application of Bi-gram Dice to strings *X* and *Y*. The strength of n-grams is in the fact it has context sensitivity, but it does not have good resolution when gram size is increased [28]. Based on our preliminary results, for the dataset used in this work, Bi-gram Dice have better results than Levenshtein distance, Cosine distance, and Jaccard distance.

8 $$\begin{aligned} Similarity = \frac{2 \times (Bi-gram(X) \cap Bi-gram(Y)}{ Bi-gram(X) + Bi-gram(Y)} \end{aligned}$$

For example, the word *Abscess of external ear* contains 19 different bi-grams (ab, bs, al, rn, te, ex, of, ss, ce, ar, sc, le, na, er, xt, fe, so, es and ea). The word *Malignant otitis externa* contains 19 different bi-grams (na, ti, ig, te, ex, is, it, ot, nt, ma, al, li, er, xt, se, to, an, gn and rn). Both words have in common 7 bigrams (al, er, ex, na, rn, te, xt), so the bi-gram dice similarity is 0.37.

We used three metrics to evaluate the results: Precision, Recall, and F-Measure. These metrics were used to compare results obtained by our approach and expected results from the official mappings.

Precision is defined as the relation between correctly identified mappings and identified mapping (Formula 9).

9 $$\begin{aligned} Precision = \frac{\#IdentifedAndCorrectMappings}{\#Identified Mappings} \end{aligned}$$

Recall is defined as the relation between correctly identified mappings and those expected new official releases of mappings (Formula 10).

10 $$\begin{aligned} Recall = \frac{\#IdentifedAndCorrectMappings}{\#Correct Mappings} \end{aligned}$$

F-measure is the harmonic mean of precision and recall (Formula 11).

11 $$\begin{aligned} F-Measure = \frac{2 \times Precision \times Recall}{Precision + Recall} \end{aligned}$$

Tables 4 (SNOMED-CT and NCI Thesaurus), 5 (SNOMED-CT and ICD-9) and 6 (MeSH and ICD-10) show the obtained results in terms of precision, recall, and f-measure in applying our Algorithm 1 for the studied datasets.

Table 4 presents results for SNOMED-CT and NCI. Precision and recall increased with higher source levels. Precision increased and recall decreased with higher thresholds. Precision and recall increased between target levels 0 and 1, but they did not change for higher target levels. We found that the best f-measure results have source level 3, threshold 0.9, and target levels 2 and 3.

**Table 4:** Mapping refinement results for SNOMED-CT and NCI

Source level	Threshold	Target level	Precision	Recall	F-Measure
1	0.5	0	0.009	0.018	0.012
		1	0.042	0.101	0.060
		2	0.048	0.120	0.068
		3	0.048	0.127	0.069
	0.75	0	0	0	0
		1	0.088	0.082	0.085
		2	0.086	0.082	0.084
		3	0.101	0.108	0.104
	0.9	0	0	0	0
		1	0.344	0.070	0.116
		2	0.378	0.089	0.144
		3	0.341	0.089	0.140
2	0.5	0	0.006	0.025	0.010
		1	0.031	0.139	0.051
		2	0.035	0.171	0.058
		3	0.034	0.184	0.058
	0.75	0	0.006	0.006	0.006
		1	0.089	0.120	0.102
		2	0.105	0.152	0.124
		3	0.108	0.165	0.130
	0.9	0	0.071	0.006	0.012
		1	0.357	0.095	0.012
		2	0.423	0.127	0.195
		3	0.407	0.139	**0.208**
3	0.5	0	0.004	0.019	0.006
		1	0.023	0.139	0.039
		2	0.029	0.190	0.050
		3	0.028	0.190	0.048
	0.75	0	0.005	0.006	0.006
		1	0.08	0.127	0.097
		2	0.104	0.177	0.125
		3	0.097	0.177	0.125
	0.9	0	0.071	0.006	0.012
		1	0.356	0.101	0.158
		2	0.434	0.146	**0.218**
		3	0.418	0.146	**0.216**

Note: Reprinted from [7]

Results presented in Table 5 (concerning the mapping between SNOMED-CT and ICD-9) differ from those of SNOMED-CT and NCI. A higher source level decreases precision for thresholds 0.5 and 0.75 but did not affect the results for threshold 0.9. Recall decreased for higher source levels for threshold 0.5, but it had a minor increase with thresholds 0.75 and 0.9. A higher threshold increases precision but decreases recall. The target level decreased precision and recall between 0 and 1 for threshold 0.5 but did not affect precision and recall for higher thresholds. We found that the best results in terms of f-measure have a low threshold and low target level, but it is not affected by source level.

**Table 5:** Mapping refinement results for SNOMED-CT and ICD-9

Source level	Threshold	Target level	Precision	Recall	F-Measure
1	0.5	0	0.535	0.186	**0.276**
		1	0.340	0.163	**0.220**
		2	0.310	0.152	**0.204**
		3	0.296	0.145	0.196
	0.75	0	0.630	0.037	0.069
		1	0.461	0.044	0.081
		2	0.449	0.041	0.075
		3	0.439	0.041	0.075
	0.9	0	0.778	0.004	0.009
		1	0.692	0.006	0.011
		2	0.727	0.005	0.10
		3	0.75	0.006	0.011
2	0.5	0	0.325	0.181	**0.233**
		1	0.233	0.159	0.189
		2	0.241	0.167	0.198
		3	0.230	0.160	**0.230**
	0.75	0	0.487	0.046	0.084
		1	0.349	0.0455	0.080
		2	0.449	0.042	0.076
		3	0.382	0.048	0.085
	0.9	0	0.8	0.005	0.010
		1	0.687	0.007	0.014
		2	0.615	0.005	0.010
		3	0.615	0.005	0.010
3	0.5	0	0.256	0.177	**0.209**
		1	0.190	0.158	0.166
		2	0.200	0.159	0.177
		3	0.199	0.157	0.175
	0.75	0	0.444	0.051	0.091
		1	0.342	0.049	0.085
		2	0.360	0.050	0.089
		3	0.348	0.049	0.085
	0.9	0	0.833	0.006	0.013
		1	0.687	0.007	0.014
		2	0.687	0.007	0.014
		3	0.687	0.007	0.014

Note: Reprinted from [7]

Table 6 presents the results for the refinement of MeSH and ICD-10. Precision and recall increased when source level went from 1 to 2, but precision decreased and recall did not change with source level 3. Precision and recall increased between target levels 0 and 1 but did not change to greater target levels. Precision improved with a higher threshold and recall did not change between 0.5 and 0.75, but decreased with a threshold of 0.9. We found the best results with source level 2, threshold 0.75, and target level between 1 and 3.

**Table 6:** Mapping refinement results for MeSH and ICD-10

Source level	Threshold	Target level	Precision	Recall	F-Measure
1	0.5	0	0.059	0.048	0.053
		1	0.059	0.048	0.053
		2	0.059	0.048	0.053
		3	0.0625	0.048	0.054
	0.75	0	0.250	0.048	0.08
		1	0.250	0.048	0.08
		2	0.250	0.048	0.08
		3	0.250	0.048	0.08
	0.9	0	0	0	0
		1	0	0	0
		2	0	0	0
		3	0	0	0
2	0.5	0	0.067	0.095	0.078
		1	0.079	0.143	0.102
		2	0.0714	0.143	0.095
		3	0.0714	0.143	0.095
	0.75	0	0.250	0.048	0.08
		1	0.429	0.143	**0.214**
		2	0.429	0.143	**0.214**
		3	0.429	0.143	**0.214**
	0.9	0	0	0	0
		1	1.000	0.095	0.174
		2	1.000	0.095	0.174
		3	1.000	0.095	0.174
3	0.5	0	0.036	0.095	0.052
		1	0.044	0.143	0.067
		2	0.043	0.143	0.067
		3	0.043	0.143	0.066
	0.75	0	0.125	0.048	0.069
		1	0.333	0.143	0.2
		2	0.333	0.143	0.2
		3	0.333	0.143	0.2
	0.9	0	0	0	0
		1	1.000	0.095	0.174
		2	1.000	0.095	0.174
		3	1.000	0.095	0.174

Note: Reprinted from [7]

**Fig. 5:**
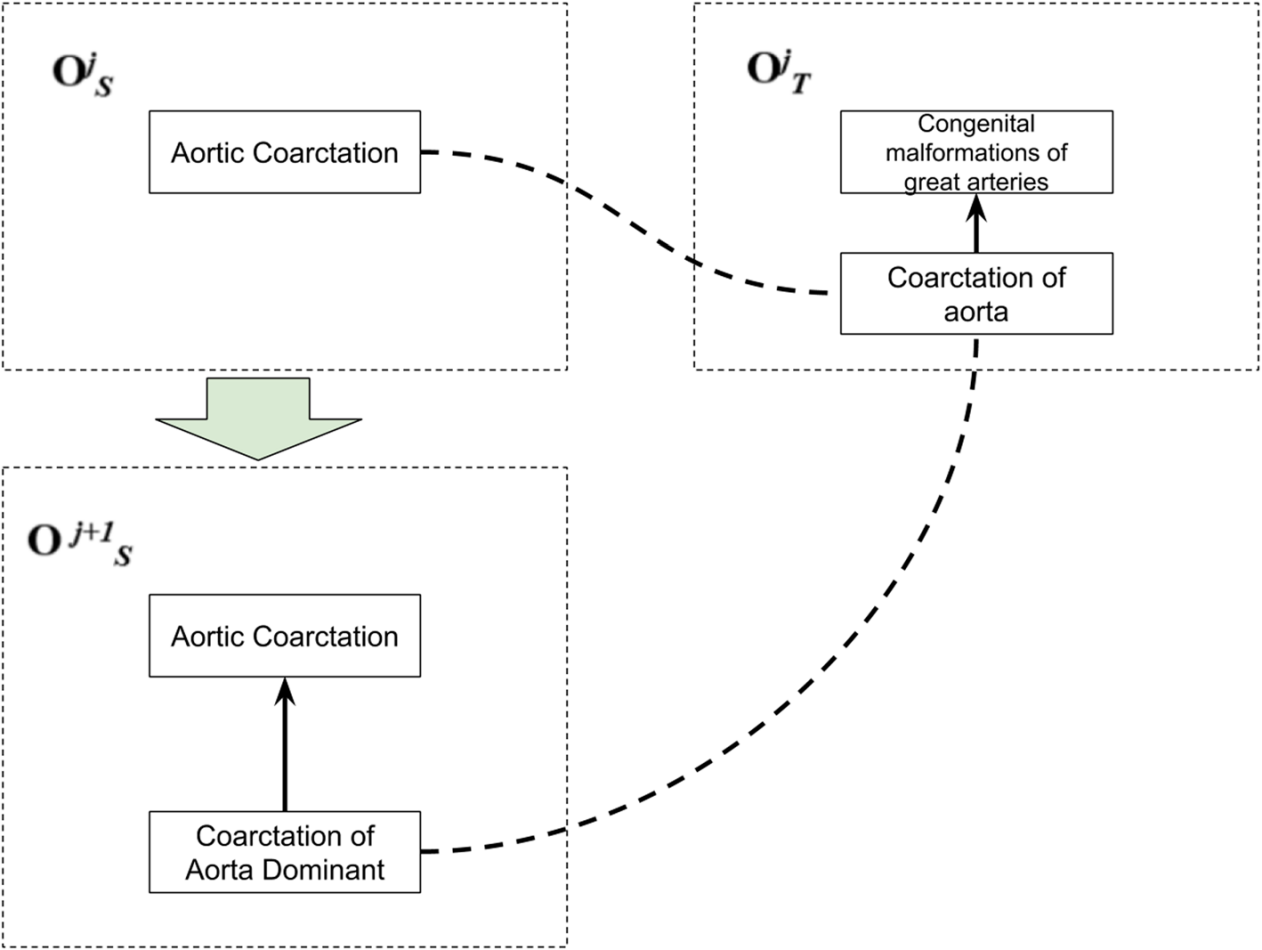
Example of a correct mapping refinement for MeSH-ICD10

Figure 5 represents a correct mapping refined from MeSH-ICD10. In this case, *Coarctation of Aorta Dominant* is the new concept added to MeSH. First, the algorithm investigated the context of the new concept and found a parent concept with mapping to the target concept (*Aortic Coarctation*).

Second, the algorithm explored the context of the target concept and found all candidate concepts. In this example, *Coarctation of aorta* and *Congenital malformations of great arteries* were candidate concepts. The similarity value between concepts was calculated as the highest string similarity between attributes of the concept. The similarity was represented as a rational number between 0.0 and 1.0. Attributes are concept properties as strings of a term denoting concepts. Concept character terms are compared using similarity measures.

*Coarctation of Aorta Dominant* and *Coarctation of aorta* presented 0.72 of similarity value. *Coarctation of Aorta Dominant* and *Congenital malformations of great arteries* presented 0.34 of similarity value. In this case, *Coarctation of aorta* had higher similarity than *Congenital malformations of great arteries*. As expected, the algorithm created a new mapping between *Coarctation of Aorta Dominant* and *Coarctation of aorta*.

**Fig. 6:**
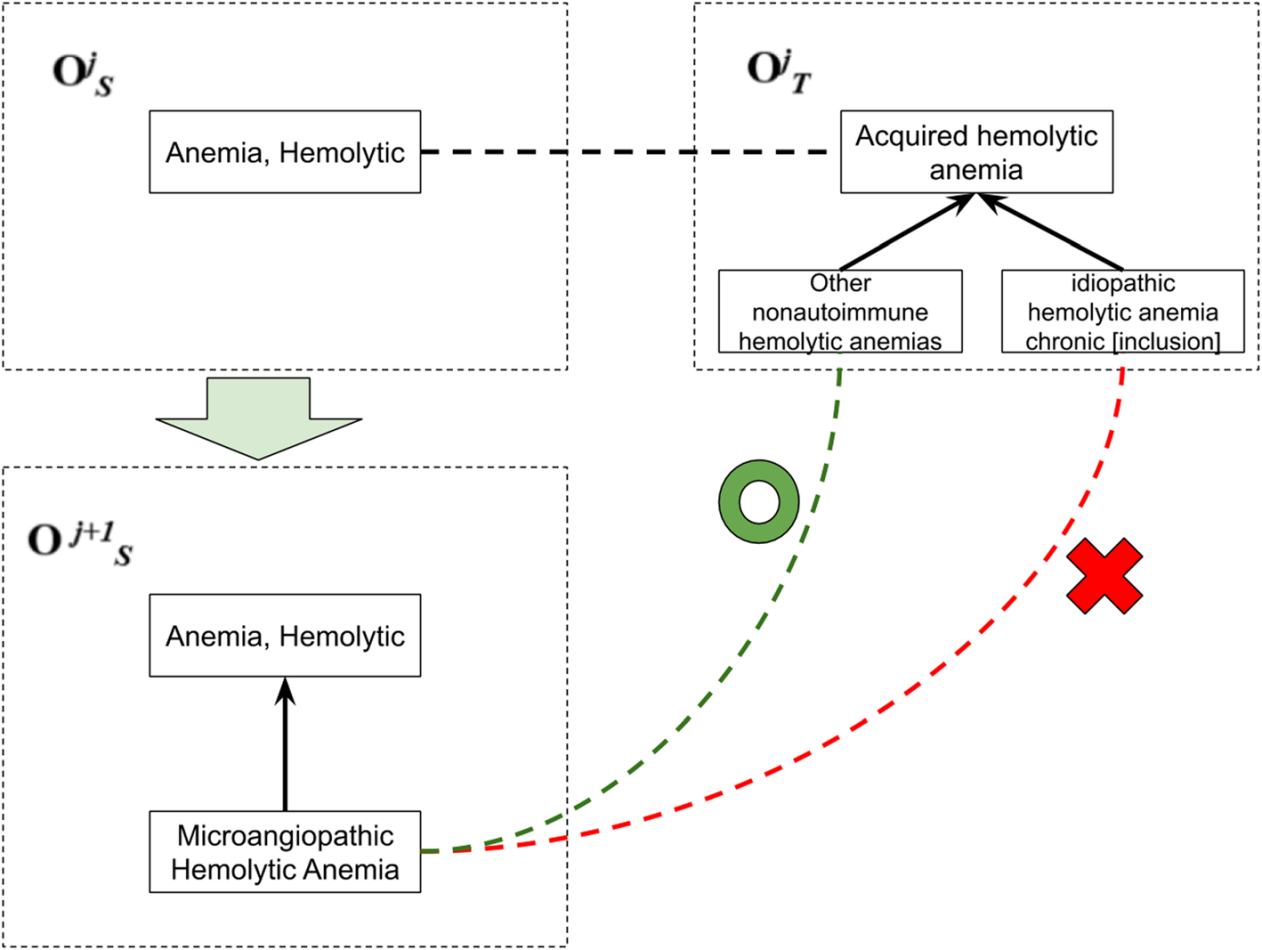
Example of a wrong mapping refinement for MeSH-ICD10

Figure 6 represents another case from MeSH-ICD10, but the algorithm created a wrong mapping. In this case, the newly added concept is *Microangiopathic Hemolytic Anemia*. The parent concept inside the context of the new concept was Anemia, Hemolytic and the candidate concepts were *Acquired hemolytic anemia*, *Other nonautoimmune hemolytic anemias* and *idiopathic hemolytic anemia chronic [inclusion]*. *Microangiopathic Hemolytic Anemia* and *Acquired hemolytic anemia* presented 0.46 of similarity value. *Microangiopathic Hemolytic Anemia* and *Other nonautoimmune hemolytic anemias* presented 0.41 of similarity. *Microangiopathic Hemolytic Anemia* and *idiopathic hemolytic anemia chronic [inclusion]* had similarity 0.61. The algorithm created a new mapping between *Microangiopathic Hemolytic Anemia* and *idiopathic hemolytic anemia chronic [inclusion]*, but the expected mapping was with *Other nonautoimmune hemolytic anemias*.This error is due to the string similarity of the terms and demonstrates how semantic similarity comparison may be beneficial to mitigate this type of error.

We evaluated our proposal in considering the neighborhood for the refinement of new mappings associated with new concepts (Algorithm 1) with the approach in applying the matching with the whole target ontology (Algorithm 2). To this end, we applied the non-context approach in the datasets considering the threshold $\tau$ yielding the best results in Algorithm 1 obtained for each dataset.

Table 7 shows the results concerning precision, recall, and f-measure obtained for each dataset using the matching with all concepts and context approach in the target ontology. The comparison of results reveals that for the dataset SCT-NCI the results using all target concepts as candidates were better. For the dataset SCT-ICD9, our context approach is better; concerning the dataset MeSH-ICD10, the approaches obtained similar results. We must consider that applying mapping candidates with the whole target ontology presents a worse run-time complexity than our contextual approach.

**Table 7:** Mapping refinement results exploring the matching with all concepts of the target ontology

		All Concepts Approach			Context Approach		
Data set	Threshold	Precision	Recall	F-Measure	Precision	Recall	F-Measure
SCT-NCI	0.9	0.593	0.525	**0.557**	0.434	0.146	**0.218**
SCT-ICD9	0.5	0.264	0.042	**0.072**	0.525	0.186	**0.276**
MeSH-ICD10	0.75	0.312	0.238	**0.270**	0.429	0.143	**0.214**

Note: Adapted from [7]

### Analysing old ontology version to refine mappings

#### Mapping refinement to obtain $\mathcal {M}^{\prime{j}}_{ST}$ (“looking at the past to refine mappings”)

We applied our defined Algorithm 3 to invoke the appropriate refinement actions based on the additions $c_s \in LOINC_{en}^{2.65}$ participating in the alignment. Our ontology matching system employed the Levenshtein edit-distance [29] as a similarity measure, aided by automatic translation from Spanish to English by the Google Translate API. The automatic translation was required to enable the comparison of the label of entities in the same language (in this case, the English language). Google Translate API was chosen because among the web translation services, *Google Translate* has the best results in the context of concept mapping, surpassing other web services [30].

Our results present real-life examples of the outcome in applying our technique to LOINC entities. Table 8 presents the results as the effect of applying the refinement actions. The original input mapping set $\mathcal {M}^{2.65}_{en-es}$ contains 54599 correspondences. In the last version update, from release version 2.64 to 2.65, over 8000 entities have suffered some change, with 1408 entities being newly added. All of these entities were involved as part of mappings, thus allowing the application of the **AdditionProcedure**. The technique refined the mapping set and the refinement actions performed generated 1513 new semantically enriched mappings, by increasing the number of mappings to a total of 56113.

**Table 8:** Evaluation results for mapping refinement in LOINC

Input mapping size	Total of newly added concepts	Total of changes affecting mappings	Mapping size after applying refinement actions
54,599	1,408	1,408	56,113

Note: Reprinted from [6]

**Fig. 7:**
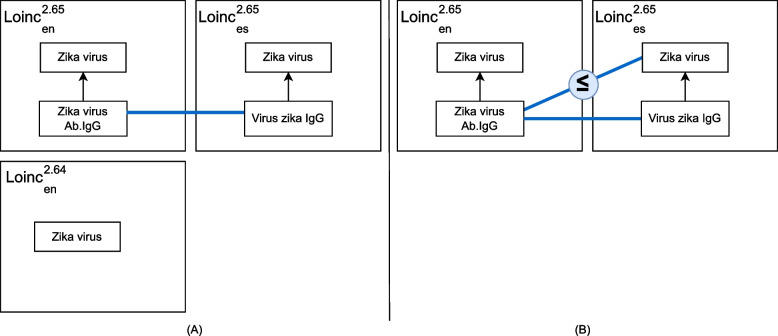
**A** Addition of the concept *Zika virus Ab.IgG* from $LOINC^{2.64}_{en}$ release version to $LOINC^{2.65}_{en}$. **B** Resulting mapping set after refinement action application

Figure 7(A) presents an example of a concept added during the ontology evolution from the release version 2.64 to 2.65 of LOINC. Concept *Zika virus Ab.IgG* is added as a sub-concept of *Zika virus* concept.

The refinement of the candidate mapping set relies on the similarity values computed between the concept *Zika virus Ab.IgG* and the concepts of the neighborhood of the target concept *Virus Zika IgG*. To this end, the algorithm 3 performed a local rematch defined in step 2. As a result of this operation, the algorithm applied a refinement action *deriveT* and derived a mapping between *Zika virus Ab.IgG* and *Virus zika* (*cf*. Fig. 7(B)).

**Fig. 8:**
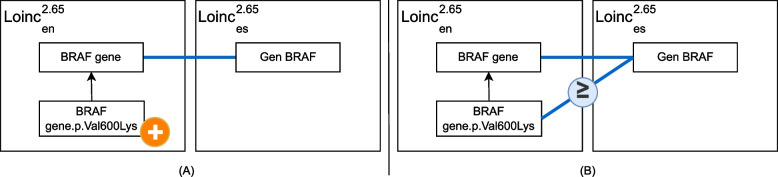
**A** Addition of the concept *BRAF gene.p.Val600Lys* from $LOINC^{2.64}_{en}$ release version to $LOINC^{2.65}_{en}$. **B** Resulting mapping set after refinement action application

Similarly, Fig. 8 presents a concept added only to source ontology, resulting in application of the refinement action *deriveS* to the candidate mapping set. This derives a new mapping with a different source concept *BRAF gene.p.Val600Lys* and the same target concept *Gen BRAF*.

## Discussion

This work addressed ways of refining existing alignments between life science ontologies. We considered different perspectives in addressing this challenge by analyzing new ontology versions to align newly added concepts; and by analyzing previous ontology version releases to execute mapping refinement actions. Our approach considered local rematch considering the context (neighbor) of concepts to be aligned.

### Discussion of mapping refinement for aligning new added concepts

This investigation aimed to create mappings to update ontology alignments based on newly added concepts in novel ontology releases (Section “Analysing new ontology version to refine mappings”). This research found that it can exploit existing mappings for mapping refinement based on newly added concepts. Our findings indicated the possibility of reaching mapping refinement for the alignment of new concepts in ontology evolution without applying a matching operation in the whole target ontology. We found further impacts in considering the level of the source concept than in the target ontology for the effectiveness of the ontology alignment refinement.

Our approach considered three variables affecting mapping quality: threshold, target level, and source level. In our results, the threshold increased precision but decreased recall. It is caused by the fact that a high threshold can remove false positive mappings, but as an effect removes correct mappings. For two datasets (SCT-NCI and MeSH-ICD10), the increase in precision compensated for the decrease in the recall. However, we observed for one dataset (SCT-ICD9) that a high threshold implied bad effects.

The use of the target level increases candidate concepts for mapping by increasing the context in the target ontology. In this sense, each newly added concept has more options of concepts in the target ontology to compare. The increase in candidate concepts enables more chances to find the correct mappings, but it can lead to obtaining wrong mapping when a wrong concept has better results in terms of similarity value than the expected concept. For two datasets (SCT-NCI and MeSH-ICD10), precision increased between target levels 0 and 1, and recall improved when the target level increased. For one dataset (SCT-ICD9), precision decreased between target level 0 and 1 for threshold 0.5, but it did not have effects on higher thresholds. The recall had only minor effects caused by changes in the target level. The results concerning the target level were very dependent on the characteristics of the datasets.

Source level increases source context to find candidate concepts from the target ontology. Our approach relies on the fact that the neighborhood of a new concept contains concepts presenting a mapping in a prior version of the ontology. If the source level is low, newly added concepts have fewer chances to find concepts mapped in a prior version. In the worst case, if no concept is mapped in a prior version, new concepts are not analyzed to find new mappings. For concepts presenting candidate concepts for lower source level, increasing source level presents the same effect as the target level. A higher source level adds more candidate concepts and increases the chances of finding a correct mapping, but it can cause more false positive mappings. In the study of the dataset SCT-NCI, higher source levels increased precision and recall. For SCT-ICD9, a higher source level achieved worse precision and recall for a lower threshold, but it was not affected by a higher threshold. For MeSH-ICD10, precision and recall increased between 1 and 2, but precision decreased for source level 3. We found that the benefits of increasing the source level depend on the threshold used for a specific dataset.

The analysis of results obtained by the all concepts approach indicated that SCT-NCI got better results using this approach. Whereas precision presented very similar results, the recall was very low using the contextual approach. SCT-ICD9 presented better results using a contextual approach. In this case, precision had good values using the contextual approach, but f-measure suffered with low recall. Findings on the dataset MeSH-ICD10 presented similar results for both approaches.

In summary, the contextual approach implied a better precision, but the all concepts approach obtained a better recall at the price of computational costs (it must examine the whole target ontology). Our future work will involve the study of additional datasets and how to obtain adequate parameters according to different ontology characteristics under analysis. Our approach to refine ontology mappings based on the contextual information would not properly work if we were unable to obtain ontology changes (actually to compute the newly added concepts) and or if we were unable to access and navigate the ontology concepts related to the new added concept. Our solution applies to other fields out of the life sciences. We only need access to the existing ontology mappings in place and compute the newly added concepts from one version to another new version of the ontology. The concepts in these ontologies need to be characterized by string terms denoting their meaning.

### Discussion of mapping refinement based on the observation of the old version of newly added concepts

This study assessed to which extent ontology evolution can be useful to decide on the application of mapping refinement actions and improve the mapping quality outcome. Our main goal with this research was to assess the usefulness of evolution change information in mapping refinement by verifying if the semantic relationships in mappings are expanded beyond equivalence meaningfully. To the best of our knowledge, the use of ontology change operations for mapping refinement has never been addressed in the literature. This aspect refers to the key originality of our method. We demonstrated the usefulness of ontology changes to aid the process of ontology mapping refinement in a case of aligned biomedical ontologies. Our study has further focused on the operation of new concept addition.

The procedure used similarity measures for a local rematch. The selection of the applicable similarity measure depends on the addressed problem because similarity measures depend on background knowledge, such as semantic similarity measures relying on semantic networks of a specific domain. In our experimental evaluation, we chose a simple, widely used, and domain-neutral similarity measure. Nevertheless, any similarity measure appropriate for the problem can be used.

The refinement procedure enriches the candidate mapping set with semantic context through predefined actions. The enrichment is beneficial for ontology merging and, as a result, for system integration.

The developed techniques reached mapping refinement without applying a matching operation to the entire ontologies involved. In addition, our technique enables the update of the semantic relation of mappings. The current approach focuses only on *is-a* and *part-of* relationships. Other relationship types will be addressed in future work.

The most prevalent approaches for refinement in the literature rely on the use of external sources. The main advantage of using evolution information is the possibility of refinement without needing an external resource. This is particularly useful when external resources are unavailable to refine the task.

The use of ontology change operations for mapping refinement purposes is limited to mappings with at least one participant ontology with multiple versions available to calculate history changes, or a list of updates between ontology versions must be available. Our proposed procedure depends on the set of ontology changes. Thus, only mappings with entities associated with ontology change(s) are eligible for the procedure. This limits the number of mappings that can be refined with this technique. The precise correctness of the generated output will be evaluated in future work by the contribution of domain experts.

## Conclusion

Ontology mappings play a central role in semantic data integration in the software systems of the biomedical domain. The result of mapping refinement increases the usefulness of mapping sets, benefiting the semantic data integration of systems. Domain knowledge update leads to new concepts in ontology versions. This requires maintaining mapping sets up-to-date according to the knowledge dynamics. We proposed two techniques to refine ontology alignments based on evolving ontologies. Our constructed algorithms considered the context of concepts as a way to find matching between concepts. Our solution provides novel and innovative research results on how to update ontology mappings on the basis of ontology changes over time. Experimental evaluation with aligned real-world biomedical ontologies demonstrated the effectiveness of our approach. Future work involves further investigating heuristics to update the type of semantic relation in the refinement procedure and include domain specialists to evaluate the correctness of the proposed concept mappings and their specific type of semantic relation.

## Data Availability

Source code and data not available.

## Abbreviations

UMLS: Unified Medical Language System
CT: Context of concept
SCT: Systematized Nomenclature of Medicine-Clinical Terms
MeSH: Medical Subject Headings
ICD9: International Classification of Diseases, Ninth Revision, Clinical Modification
ICD10: International Classification of Diseases, Tenth Revision, Clinical Modification
OWL DL: Web Ontology Language Description Logic
NCI: National Cancer Institute
LOINC: Logical Observation Identifiers Names and Codes

## Footnotes

1 As of 2023/09/12 13:09:09.

2 https://www.nlm.nih.gov/

3 https://ncit.nci.nih.gov/ncitbrowser/

4 https://www.nlm.nih.gov/research/umls/mapping_projects/icd9cm_to_snomedct.html

5 http://www.chu-rouen.fr/cismef
